# Introduction to the Toxins Special Issue on LC-MS/MS Methods for Mycotoxin Analysis

**DOI:** 10.3390/toxins9100325

**Published:** 2017-10-16

**Authors:** Aldo Laganà

**Affiliations:** Department of Chemistry, Università di Roma “La Sapienza”, Piazzale Aldo Moro 5, 00185 Rome, Italy; aldo.lagana@uniroma1.it; Tel.: +39-06-4991-3679

Various filamentous fungi can produce secondary metabolites, whose biochemical significance in fungal growth and development has not always been fully clarified; however, some of these metabolites can cause deleterious effects on other organisms and are classified as mycotoxins. The main mycotoxin-producing fungi belong to species of *Fusarium*, *Aspergillus*, *Penicillium*, *Claviceps* and *Alternaria* genera. Mostly, human exposure to mycotoxins occurs through the intake of contaminated agricultural products, or indirectly through the intake of residues or metabolite products present in foods of animal origin. Seldom, exposure may also occur through dermal contact and inhalation. The known mycotoxins belong to heterogeneous chemical classes, and they can exert highly diverse toxic effects (e.g., cancerogenic and immunosuppressive effects). 

Immunochemical methods (such as ELISA) can be used for rapid screening of mycotoxin presence; however, for confirmation purposes, analytical methods based on high-performance liquid chromatography (LC) are preferred, especially when coupled with tandem mass spectrometry (MS/MS), which allows the determination of multiclass mycotoxins in a single analysis. Moreover, the technical innovations available in LC-MS/MS instrumentation are prompting its application in monitoring the presence of contaminants in food and feed. The aim of this Special Issue is to explore the capabilities of LC-MS/MS methods in mycotoxin investigation. 

The topic of LC-MS/MS multi-mycotoxin determination is addressed in most papers. Indeed, as reported by De Santis et al. [1], due to the actual probability that co-occurring mycotoxins are present in a food or feed product, nowadays, the availability of reliable, sensitive, and versatile multi-mycotoxin methods is increasingly important; moreover, there is a wide range of matrices susceptible to mycotoxin contamination. For these reasons, the authors validated a multi-mycotoxin and multi-matrix LC-MS/MS-based method of overcoming specific matrix effects (no sample clean-up was performed after acetonitrile-water extraction) and analyzing complex cereal-based samples. The investigated mycotoxins were aflatoxin B1 (AFB1), ochratoxin A (OTA), deoxynivalenol (DON), fumonisin B1 (FB1), zearalenone (ZEN), T-2 and HT-2 toxins. 

In this special issue, Kim et al. [2] proposed an LC-MS/MS method for the simultaneous determination of 13 mycotoxins, namely DON, nivalenol (NIV), 3-acetylnivalenol, the main four aflatoxins (AFB1, AFB2, AFG1, and AFG2), FB1, fumonisin B2 (FB2), T-2, HT-2, ZEN, and OTA, in cereal grains. After the classical acetonitrile-water mixture extraction step, the sample was cleaned up by a single immunoaffinity column. In the survey of samples from South Korea, DON, NIV and ZEN were more frequently and concurrently detected in all cereal grains than OTA and AFs.

To make mycotoxin analysis more environmentally friendly, Breidbach [3] extracted DON, T-2, HT-2, and ZEN by replacing acetonitrile by ethyl acetate, and forgoing sample extract clean-up, thus minimizing solvent consumption. The LC-MS/MS method was validated in-house and through a collaborative study. The extraction was also adapted to a quick screening of AFB1 in maize by flow-injection—MS. 

A method based on the QuEChERS (quick, easy, cheap, effective, rugged, and safe) purification followed by LC-MS/MS analysis was optimized by Sun et al. [4] for the simultaneous quantification of 25 mycotoxins in cereals. In this case, aside from the classically investigated mycotoxins, sterigmatocystin (STC), verruculogen, enniatins, beauvericin, gliotoxin and other were also included.

Eight laboratories performed an interlaboratory study to validate an LC-MS/MS method for the simultaneous determination of AFs and STC in white rice and sorghum [5]. Sample purification was carried out by solid-phase extraction. The validated method was successfully applied for the determination of AFs and STC in 20 white rice and 20 sorghum samples collected from Korean markets.

Mycotoxins can undergo modification in plants, leading to the formation of a large number of possible modified forms, whose toxicological relevance and occurrence in food and feed is still largely unexplored. The analysis of modified mycotoxins by LC-MS remains a challenge because of their chemical diversity, the large number of isomeric forms, and the lack of analytical standards. Therefore, Righetti et al. [6] explored and reviewed the potential benefits of high-resolution (HR) and ion mobility MS as a tool for separation and structure confirmation of modified mycotoxins. In fact, the analytical potential of high resolving power, accurate mass, and acquisition in full scan permits a retrospective analysis using extensive databases of hundreds of analytes and enabling the investigation of ‘newly discovered’ mycotoxins in the data of previously-analyzed samples. However, applications of ion mobility spectrometry in the separation and structure confirmation of mycotoxins have not been explored adequately so far, even though it offers great potential for gaining insight into the formation and characterization of new modified forms. 

The use of ^13^C-assisted LC-HRMS for the first comprehensive study on the biotransformation of HT-2 and T2- in oats is described by Meng-Reiterer et al. [7]. Using this approach, 16 HT-2 and 17 T-2 metabolites were annotated, including novel glycosylated and hydroxylated forms of the toxins, hydrolysis products, and conjugates with acetic acid, putative malic acid, malonic acid, and ferulic acid. Further targeted quantitative analysis was performed to study toxin metabolism over time, as well as toxin and conjugate mobility within non-treated plant tissues. The results show that the combination of untargeted and targeted analysis is well suited to the comprehensive elucidation of mycotoxin metabolism in plants.

In this Special Issue, a survey on a very large sample set is also included. More than 1900 samples were analyzed for toxins and metabolites by Kovalsky et al. [8], and up to 68 metabolites were found in a single sample. Co-occurrence of regulated toxins was frequent with e.g., enniatins, and moniliformin. Results indicate that considerably more than 25% of agricultural commodities could be contaminated with mycotoxins, as suggested by FAO, although this is at least partly due to the lower limits of detection in the current survey. Observed contamination ranged from 7 to 79% for B trichothecenes and 88% for ZEN.

*Alternaria* toxins (ATs) are usually less frequently studied; nevertheless, some papers about them are also presented in this collection. The formation of ATs depends on the species and complex interactions of various environmental factors, and is not fully understood. Therefore, Zwickel et al. [9] studied the influence of temperature, substrate, and incubation time on the production of thirteen ATs and three sulfoconjugated ATs by three different Alternaria isolates. 

Rodríguez-Carrasco et al. [10] developed an analytical method based on LC-MS/MS detection for the simultaneous quantification of the three main ATs in tomato and tomato-based products. The extraction was performed by dispersive liquid-liquid microextraction (DLLME). Mycotoxins were efficiently extracted from sample into a droplet of chloroform by DLLME technique using acetonitrile as a disperser solvent. 

The possible exposure to four ATs from grain and grain-based products has been studied by Xu et al. [11]. A total of 370 freshly harvested wheat kernel samples collected in China were analyzed. 

I hope that this Special Issue, constituted by ten research articles and one review, will provide readers an overview about the multi-mycotoxin analysis and new insights on the investigation of emerging and modified mycotoxins. In particular, the identification of modified mycotoxins and mycotoxin metabolites could take advantage of the unique identification features of HR MS.
